# Gender and myocardial fibrosis by CMR are independent predictors of myocardial dysfunction in patients with Chagas' heart disease

**DOI:** 10.1186/1532-429X-17-S1-O79

**Published:** 2015-02-03

**Authors:** Antonildes N Assunção, Bernardo B Lopes, Alejandra Villanueva, Liliane Rocha, Carolina S Reiser, Eduardo B Falchetto, Simone Cristina S Costa, Barbara Ianni, Fabio Fernandes, Marly U Lopes, Roberto Kalil, Carlos E Rochitte

**Affiliations:** 1 Heart Institute, InCor, University of Sao Paulo Medical School, Sao Paulo, Brazil; 2 Felicio Rocho Hospital, Minas Gerais, Brazil

## Background

Chagas' heart disease (CHD) is a common cause of non-ischemic cardiomyopathy in Latin America. In CHD, male gender has been shown to have worse clinical prognosis, but the reasons for that remain unclear. Cardiovascular magnetic resonance (CMR) imaging is able to assess the extent of myocardial fibrosis (MF), which correlates to left ventricle ejection fraction (LVEF), and is a marker of disease severity. Our objective was to investigate whether male gender is associated to greater myocardial injury, assessed by CMR as myocardial fibrosis and dysfunction.

## Methods

We retrospectively analyzed 139 patients with CHD of 3 previous CMR studies from 2 different sites. Patients had CMR examination on 1.5 T MRI systems with cine-MR with SSFP sequence for left ventricle function evaluation and late gadolinium enhancement for myocardial fibrosis detection. LV volumes and function were measured by Simpson's method. All analyses were performed using CVi42 software (Circle CVi, Calgary, CA). χ^2^, Fisher exact tests, t test and Mann-Whitney test when appropriate and for correlation Spearman test, were performed using Stata 12.

## Results

LVD was present in 45.3% of patients and was more frequent in male than in female (32.9% vs. 67.1%, p<0,001). The presence of MF was greater in male than in female group (42.4% vs. 57.6%, respectively, p<0,001). Quantified MF was significantly higher in males (16.8% vs. 7.4%, p<0.001). Functional class (FC-NYHA) was also significantly greater in males (Table 1 and 2). There was a good and significant negative correlation between LVEF and MF (r=-0.69, p<0.001). A multivariate model using logistic regression showed that gender and MF are independent predictors of LVD, with an OR=6.17 for males (p=0.02) and 1.27 (p<0.001) for MF.

**Table 1:** Characteristics of patients with CHD by myocardial dysfunction.

	All (n=139)	LVEF ≥ 50 (n=63)	LVEF < 50 (n=76)	P-value
Age, mean ± SD	53.6 ± 12.4	52.9 ± 13.0	54.2 ± 11.9	0.54

Male, n (%)	76 (54.6)	12 (19,5)	51 (67,1)	<0.001

LVEF, % ± SD	46.6 ± 17.6	62.7 ± 6.9	33.3 ± 11,4	<0.001

FC-NYHA, n ± SD	1.7 ± 0.9	1.0 ± 0.03	2.22 ± 0.1	<0.001

RVEF, % ± SD	46.9 ± 14.5	51.2 ± 13.6	43.1 ± 14.5	0.005

EDVI, ml/m2 ± SD	94.8 ± 47.3	65.5 ± 17.1	119.0 ± 50.6	<0.001

ESVI, ml/m2 ± SD	50.4 ± 37.6	32.2 ± 16.3	65.4 ± 43.7	<0.001

Fibrosis, g ± SD	15.4 ± 22.7	2.5 ± 4.9	26.0 ± 26.0	<0.001

Fibrosis, % ± SD	11.6 ± 14.5	2.3 ± 4.4	19.3 ± 15.3	<0.001

**Table 2:** Characteristics of patients with CHD by gender.

	Female (n=76)	Male (n=63)	P-value
Age, mean ± SD	53.5 ± 12.9	53.7 ± 11.9	0.92

LVEF, % ± SD	54.9 ± 14.6	36.7 ± 15.8	<0.001

FC-NYHA, n ± SD	1.4 ± 0.6	2.1 ± 0.9	<0.001

RVEF, % ± SD	49.8 ± 13.9	43.9 ± 14.9	0.06

EDVI, ml/m2 ± SD	76.1 ± 32.6	117.4 ± 52.5	<0.001

ESVI, ml/m2 ± SD	38.3 ± 20.8	65.1 ± 47.1	<0.001

Fibrosis, % ± SD	7.4 ± 11.7	16.8 ± 15.9	<0.001

Fibrosis, g ± SD	8.0 ± 13.7	24.3 ± 27.9	<0.001

## Conclusions

In our group of 139 CHD patients, male gender was associated with higher degree of myocardial fibrosis and left ventricle dysfunction measured by CMR. Moreover, gender and myocardial fibrosis were independent predictors of myocardial dysfunction. This is the first demonstration that myocardial injury assessed by CMR as myocardial fibrosis and dysfunction is greater in male gender, which parallels the epidemiological data indicating worse clinical prognosis in males. Further studies are necessary to investigate the pathophysiology behind these findings in humans.

## Funding

N/A.

